# Quantification of Pregenomic RNA and Covalently Closed Circular DNA in Hepatitis B Virus-Related Hepatocellular Carcinoma

**DOI:** 10.1155/2013/849290

**Published:** 2013-12-19

**Authors:** Fugui Bai, Yoshihiko Yano, Takumi Fukumoto, Atsushi Takebe, Motofumi Tanaka, Kaori Kuramitsu, Nungki Anggorowati, Hanggoro Tri Rinonce, Dewiyani Indah Widasari, Masaya Saito, Hirotaka Hirano, Takanobu Hayakumo, Yasushi Seo, Takeshi Azuma, Yonson Ku, Yoshitake Hayashi

**Affiliations:** ^1^Center for Infectious Diseases, Kobe University Graduate School of Medicine, Kobe 650-0017, Japan; ^2^Department of Gastroenterology, Kobe University Graduate School of Medicine, Kobe 650-0017, Japan; ^3^Department of Hepato-Biliary-Pancreatic Surgery, Kobe University Graduate School of Medicine, Kobe 650-0017, Japan

## Abstract

Pregenomic RNA (pgRNA) is generated from covalently closed circular DNA (cccDNA) and plays important roles in viral genome amplification and replication. Hepatic pgRNA and cccDNA expression levels indicate viral persistence and replication activity. This study was aimed to measure hepatic pgRNA and cccDNA expression levels in various states of hepatitis B virus (HBV) infection. Thirty-eight hepatocellular carcinoma (HCC) patients, including 14 positive for hepatitis B surface antigen (HBsAg) and 24 negative for HBsAg but positive for anti-hepatitis B core (anti-HBc) antibody, were enrolled in this study. In HBsAg-negative but anti-HBc-positive group, HBV-DNA was detected in 20 of 24 (83%) noncancerous liver tissues for at least two genomic regions based on polymerase chain reaction (PCR) analysis. pgRNA and cccDNA expression levels in occult HBV-infected patients were significantly lower than those in HBsAg-positive patients (*P* < 0.001). pgRNA and cccDNA in cancerous tissues were also detected without significant difference from those in noncancerous tissues. In conclusion, cccDNA and pgRNA are detected and represented HBV replication not only in noncancerous but also in cancerous liver tissues. In addition, the replication is shown in not only patients with HBsAg-positive but also occult HBV-infected patients, suggesting the contribution to HCC development.

## 1. Introduction

Hepatitis B virus (HBV) infection remains a major health problem, even though methods for preventing vertical transmission and treatment guidelines have been introduced. Approximately, 2 billion people worldwide are infected with HBV. Three hundred fifty million of them have chronic infection. HBV is particularly endemic in sub-Saharan Africa, the Pacific, and Asia [1]. It frequently causes chronic hepatitis, which, subsequently, progresses to liver cirrhosis and hepatocellular carcinoma (HCC). HBV is responsible for most of the worldwide attributable risk of liver cirrhosis and HCC. The risk is even higher in endemic regions [2].

HBV is an incomplete double-stranded circular DNA virus, with a DNA length of approximately 3,200 bp. After HBV infects the liver cells, the relaxed-circular DNA is transferred into the nucleus and is repaired to covalently closed circular DNA (cccDNA) by DNA polymerase. The cccDNA plays a key role in the life cycle of the virus. It acts as a template for the generation of mRNAs, including pregenomic RNA (pgRNA) [3–5]. The transcription of cccDNA generates four kinds of mRNA: PreC/C (3.5 kb), PreS1 (2.4 kb), S (2.1 kb), and X (0.7 kb). The transcription of cccDNA is a critical step for viral genomic amplification and replication. PgRNA is also generated during the transcription from cccDNA and works as a template of viral genomic DNA.

The cccDNA is detected in various phases of chronic hepatitis, and its levels reflect viral replication in the infected cells. HBV-DNA is sometimes detected in patients negative for hepatitis B surface antigen (HBsAg) but positive for anti-hepatitis B core (anti-HBc) antibody and/or anti-hepatitis B surface (anti-HBs) antibody, which is often called occult HBV infection [4]. Recent studies revealed that cccDNA was expressed in HCC without HBs antigen [5]. However, it is still debated whether HBV-DNA is responsible for the development of HCC in patients with occult HBV infection.

In this study, we measured the levels of cccDNA and pgRNA in cancerous and contiguous noncancerous liver tissues of HCC patients with overt and occult HBV infection to examine the viral existence and replication. We also determined the nucleotide variations and amino acid mutations in patients with occult HBV infection.

## 2. Materials and Methods

### 2.1. Patients

Cancerous and contiguous noncancerous liver tissues were obtained from 38 HCC patients (mean ± SD, 63.37 ± 12.01 years; male-female ratio, 34/4) who underwent surgical resection at Kobe University Hospital, Japan, between April 2010 and December 2011. HBsAg-positive carriers (*n* = 14) and HBsAg-negative/HBcAb-positive patients (*n* = 24) were enrolled in this study. Hepatitis C virus (HCV) infected patients who were detected by anti-HCV positivity were excluded. Tissue samples were rapidly frozen in liquid nitrogen and stored at –80°C after resection.

The demographic and clinical data, including age, sex, body mass index (BMI), results of standard test for HBV-related HCC, and stage of the disease, were collected from medical record after institutional review board approval was obtained.

Written informed consent was obtained from each patient before all the procedures in this study were done. This study was reviewed and approved by the Ethics Committee at Kobe University.

### 2.2. Detection of HBV-DNA, cccDNA, and pgRNA in Tissue Samples

Total DNA and RNA from cancerous and contiguous noncancerous tissues were extracted from about 20 mg of liver tissues using a QIAamp DNA Mini Kit (QIAGEN Sciences, Germantown, MD) and Isogen (Nippon Gene, Tokyo, Japan). The DNA and RNA concentrations were measured spectrophotometrically. The cDNA was synthesized from five *μ*g of total RNA using the Oligo (dT) 15 primer (Promega, Madison, WI) and M-MLV Reverse Transcriptase (Invitrogen, Carlsbad, CA) according to the manufacturers' instructions.

To detect intrahepatic HBV-DNA, a nested polymerase chain reaction (PCR) was carried out using the specific primers for amplifying S, precore-core, polymerase, and X region. The primers used are listed in Table 1. The first and second round PCRs were performed under the same condition described previously [5]. Appropriate controls were included in each PCR. The PCR products were resolved on 2% agarose gels and visualized under ultraviolet illumination with ethidium bromide staining. Patients, who were serologically negative for HBsAg but positive for intrahepatic HBV-DNA at least in two regions of HBV genome based on PCR assays, were defined as having occult infections.

We further performed quantitative real-time PCR using an Applied Biosystems 7500 RT-PCR System (Applied Biosystems, Foster City, CA). Briefly, 2 *μ*L of HBV-cccDNA or pgRNA was amplified using TaqMan Gene Expression Master Mix (Applied Biosystems, Foster City, CA), primers for cccDNA (CCC and BC1) or pgRNA (PGP and BC1), and FRET hybridization probes (hbvLC and hbvFL) as listed in Table 1. The amplification was performed under conditions described previously [8].

To avoid cross-contamination between samples, standard precautions were used for all procedures. Reagents, samples, and amplified products were stored in separate areas.

### 2.3. Determination of HBV Genotypes and Subgenotypes

HBV genotypes and subgenotypes were determined by phylogenetic analysis based on the S region. Part of S region was amplified in PCR using HB2F and HB2R primer (Table 1) with condition described previously [7]. The amplified fragments were purified using ExoSAP-IT (USB, Cleveland, OH) and directly sequenced using BigDye Terminator v3.1. Cycle Sequencing Kit (Applied Biosystems, Foster City, CA). The sequencing was performed in an ABI PRISM 3100-*Avant* Genetic Analyzer (Applied Biosystems, Foster City, CA). The sequences were edited manually and subsequently aligned using Clustal X version 2.0.12 software (http://www.clustal.org/). A phylogenetic tree was constructed by the neighbor-joining method using the Molecular Evolutionary Genetic Analysis (MEGA) version 4.0.2 software (http://megasoftware.net/) [9]. To show the reliability of this analysis, bootstrap resampling and reconstruction were performed 1,000 times [10].

### 2.4. Detection of Amino Acid Substitutions in the “a” Determinant Region and Mutations in the Core Promoter and Precore Region

Some amino acid substitutions in the “a” determinant region are thought to result in detectable HBV-DNA in HBsAg-negative patients. Therefore, the sequences within this region were aligned and analyzed to identify amino acid substitutions among HBV strains obtained from patients positive for HBV-DNA. We used the previously described primer pairs as shown in Table 1 [7] to amplify the “a” determinant region. Core promoter and precore region were also amplified using primers and condition described previously (Table 1) [7].

Those amplified fragments were directly sequenced, and the obtained sequences from each region were aligned with reference sequences retrieved from GenBank databases to identify the substitutions or mutations.

### 2.5. Statistical Analyses

Statistical analyses were performed using IBM SPSS Statistics version 21.0 (IBM, Armonk, NY). The categorical variables were analyzed using the *χ* ^2^ test or Fisher's exact test, whereas continuous variables were analyzed using independent *t*-tests or the Mann-Whitney test. Differences were considered statistically significant at *P* < 0.05.

## 3. Results

### 3.1. Clinical Characteristics of the Patients

The clinical characteristics of HBsAg-negative/HBcAb-positive patients were compared with those of HBsAg-positive carriers (Table 2). The HBsAg-positive carriers were significantly younger than HBsAg-negative/HBcAb-positive patients (55.7 ± 12.0 versus 68.7 ± 9.3 years; *P* = 0.002). The complication of diabetes mellitus is significantly higher in HBsAg-negative/HBcAb-positive patients. It was indicated by higher level of HbA1c in this group (5.96 ± 0.87 versus 5.04 ± 0.33; *P* < 0.001). Ten of them (42%) were treated by oral medication and/or insulin injection therapy for diabetes. In addition, four patients had massive alcohol consumption and had been diagnosed as alcoholic cirrhosis. The average body mass index (BMI) in HBsAg-negative/HBcAb-positive patients tended to be high compared with those in HBsAg-positive carriers (23.1 ± 3.4 versus 20.5 ± 3.7; *P* = 0.079) although not significant statistically. Four HBsAg-positive patients were treated by nucleotide analogue for more than one year, and HBV-DNA was undetectable in their sera. A significantly greater proportion of HBsAg-positive carriers had an HBV viral load of >4 log copies/mL as compared with HBsAg-negative/HBcAb-positive patients (42.9% versus 0%; *P* = 0.012). Thirty-two HBV strains could be genotyped. Almost all (96.9%) patients were infected by genotype C2. Only one patient (3.1%) was infected by genotype B1 (Table 2 and Figure 2).

### 3.2. Detection of HBV-DNA, cccDNA, and pgRNA in the Tissue

**Figure 1:**
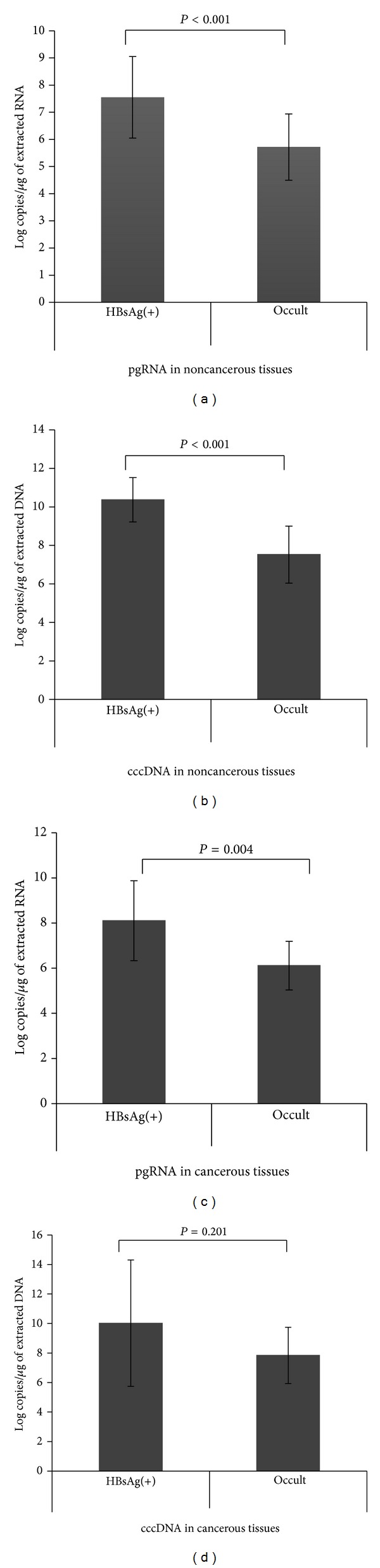
Quantitative analysis of pgRNA (a, c) and cccDNA (b, d) in noncancerous and cancerous liver tissues. The pgRNA and cccDNA expression levels were significantly higher in HBsAg-positive patients than in occult HBV-infected patients.

**Figure 2:**
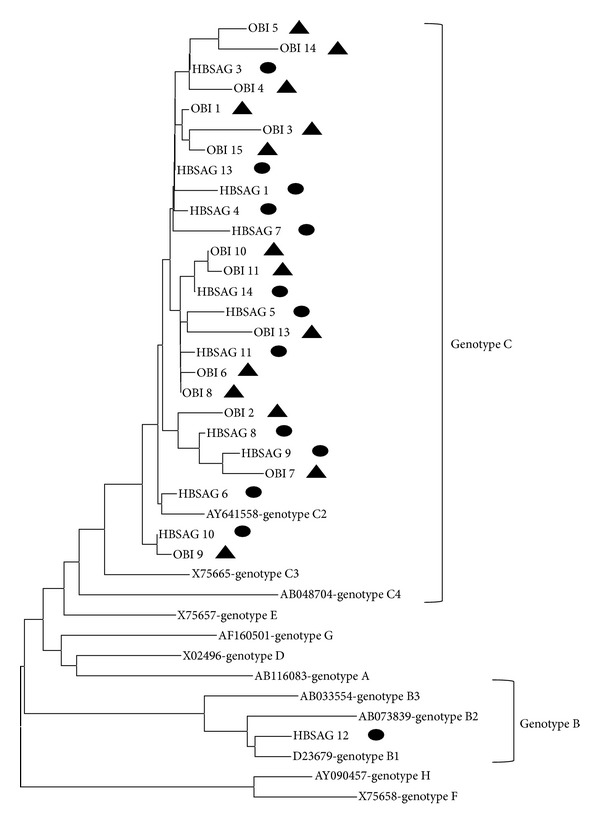
Phylogenetic tree analysis of the S gene sequences of strains isolated in this study. The HBV strains from different genotypes (A to H) are obtained from GenBank. The GenBank HBV sequences are indicated with their accession number followed by genotypes. The sequences determined in this study are indicated by isolate number, starting with HBSAG or OBI, and are labelled with black circles and triangles for HBsAg-positive patients and patients with occult HBV infection, respectively.

Based on the nested PCR analysis, HBV-DNA was detected in all cancerous and noncancerous tissues of HBsAg-positive carriers. On the other hand, at least two HBV genomic regions were detected in 20 of 24 (83%) noncancerous tissues from HBsAg-negative/HBcAb-positive patients (*P* = 0.003). Those patients were classified as having occult HBV infection. The S (71% versus 38%, *P* = 0.02) and X (88% versus 50%, *P* = 0.005) regions were detected more frequent in noncancerous tissues than in cancerous tissues (Table 3). Twenty patients with occult HBV infection were compared with 14 HBsAg-positive carriers. The mean pgRNA and cccDNA expression levels in noncancerous tissues were significantly lower in occult HBV-infected patients (5.72 ± 1.22 log copies/*μ*g of extracted RNA and 7.52 ± 1.48 log copies/*μ*g of extracted DNA, resp.) than in HBsAg-positive carriers (7.55 ± 1.50 log copies/*μ*g of extracted RNA and 10.37 ± 1.15 log copies/*μ*g of extracted DNA, resp.; both, *P* < 0.001). In addition, pgRNA and cccDNA expression levels in cancerous tissues were lower in occult HBV-infected patients (6.11 ± 1.08 log copies/*μ*g of extracted RNA and 7.84 ± 1.91 log copies/*μ*g of extracted DNA, resp.) than in HBsAg-positive patients (8.10 ± 1.77 log copies/*μ*g of extracted RNA and 10.02 ± 4.28 log copies/*μ*g of extracted DNA, resp.; *P* = 0.004 and *P* = 0.201, resp.; Figure 1). The pgRNA and cccDNA in HBsAg-positive patients were also compared in relation with nucleotide analogue therapy. However, no significant difference was detected between undertreated patients and naïve patients (pgRNA, 7.58 ± 1.48 versus 7.45 ± 1.62 log copies/*μ*g of extracted RNA; cccDNA, 10.13 ± 0.98 versus 10.15 ± 1.34 log copies/*μ*g of extracted DNA, resp.). The ratio of pgRNA/cccDNA in noncancerous tissues was also examined, but no difference was detected between HBsAg-positive patients and occult HBV-infected patients (0.75 ± 0.18 versus 0.85 ± 0.19, resp.).

### 3.3. Amino Acid Substitutions in the “a” Determinant Region and Mutations in the Core Promoter and Precore Region

Table 4 shows the distribution of amino acid substitutions in the “a” determinant region in HBsAg-positive patients and occult HBV-infected patients. Two specific substitutions (I126T and I126V) were detected in the occult HBV infection group. However, all of the occult infected patients had the viral loads less than 4 log copies/mL. Substitutions in I126T and T131P were also detected in HBsAg-positive patients.

The A1762T/G1764A, T1846A, C1858, and G1896A mutations were detected in HBsAg-positive carriers and in occult HBV-infected patients, without significant differences in their prevalence rates. However, the T1753C mutation was only found in HBsAg-positive carriers (29%), and it was statistically significant (*P* = 0.033; Table 5).

## 4. Discussion

HBV is a major cause of HCC in most Asian countries. Chronic HCV infection is the leading cause and HBV infection is the second most common cause of HCC in Japan [11]. However, approximately 10% of HCC patients were serologically negative for HBsAg and HCV-Ab [12]. Chronic forms of hepatitis, particularly alcoholic hepatitis, steatohepatitis, and autoimmune hepatitis, may induce HCC in the absence of hepatitis viral infection. The number of cases of HCC without hepatitis viral infection has increased in recent years because of the introduction of vaccination programmes aimed at preventing vertical transmission of HBV and screening systems to prevent HCV infection from transfusion and transmission [13].

Occult HBV infection is diagnosed when serum is negative for HBsAg, but serum or liver is positive for HBV-DNA, regardless of anti-HBc status [14]. Several studies have assessed HBV-DNA status in HBsAg-negative HCC patients [6, 15]. However, it is still controversial whether occult HBV infection is associated with liver damage. In particular, although it was reported that occult HBV infection did not progress to severe liver disease [16], other studies showed that occult HBV infection caused liver disease progression in patients with chronic HCV infection [17].

Earlier studies suggested that individuals could recover from self-limited acute hepatitis because of the role of cytotoxic or memory T cells. However, these patients might carry the HBV genome for a long time without showing clinical signs of hepatic injury [18]. Furthermore, histopathological examination of such patients showed mild necroinflammation after decades of recovery [19]. In the immunocompetent state, occult HBV is not destructive, but progressive liver dysfunction may occur if other deleterious factors are present [20]. In this study, the HBsAg-negative/HBcAb-positive patients were significantly older than the HBsAg-positive carriers, suggesting that the hepatocarcinogenesis between these two groups was different.

The association of occult HBV infection with HCC has been extensively reviewed [21]. It was also reported that cryptic HBV infection had a pro-oncogenic role in chronic HCV carriers and in cryptogenic liver diseases [6]. In animal studies, for woodchucks and ground squirrels infected with woodchuck or ground-squirrel hepatitis virus, liver cancer progression was apparent, even after elimination of these viruses [22]. A previous study showed that occult HBV was strongly associated with liver cancer, independently of age, sex, cirrhosis, and contemporary HCV infection. Aside from indirect oncogenic mechanisms (e.g., mild necroinflammation), direct oncogenic mechanisms, including viral integration into the host genome and maintenance of transcriptional activity, allow the production of potential pro-oncogenic factors, such as X protein, which may contribute to the development of cirrhosis and liver cancer [6].

In the present study, cccDNA and pgRNA in cancerous tissues were higher than those in noncancerous tissues, and this was supported by previous data [5]. However, several reports showed that cccDNA levels in cancerous tissues were lower than those in noncancerous tissues [23]. The controversial result might be dependent on the difference of the necroinflammation and liver fibrosis [24]. It was also reported that cccDNA in the liver was correlated with serum HBV-DNA viral load and decreased during antiviral therapy [25]. In the present study, four HBsAg-positive patients were treated by nucleotide analogue therapy more than one year. However, cccDNA was not different regardless of therapy. It might be partly caused by sample number and therapeutic duration.

This study revealed that intrahepatic cccDNA and pgRNA levels in cancerous and noncancerous tissues were low in occult HBV-infected patients and were lower than those in HBsAg-positive carriers. These results suggest that transcriptional activity was low in occult HBV-infected HCC patients. Even though transcriptional activity and replication were lower in HCC patients with occult HBV infection, it might still be sufficient to induce HCC [5].

Other studies have detected cccDNA and pgRNA in cancerous and noncancerous tissues in patients with occult HBV infection [5, 6]. HBV cccDNA is formed from relaxed circular-DNA and enters the nuclei of the host's cells. This stable nonintegrated minichromosome is used as a template for the transcription of viral mRNAs and pgRNA. In turn, viral mRNA is used to generate viral proteins and replication factors. The pgRNA is then encapsidated and reverse-transcribed into HBV-DNA. Animal experiments revealed that 1–50 cccDNA molecules gather in the nucleus, but the viral and host factors that regulate cccDNA are still poorly defined [26].

Some studies of duck hepatitis B virus revealed a negative feedback mechanism by which large surface protein suppresses cccDNA production [27]. In the present study, the S and X regions were detected in significantly more noncancerous tissues than in cancerous tissues. A previous study reported that a decrease in HBsAg expression in the cancer may contribute to an increase in intrahepatic HBV-DNA in the form of cccDNA in cancerous tissues, suggesting that viral replication is minimal within cancer cells and that the majority of intrahepatic HBV-DNA is in the form of cccDNA [6]. In the present study, the expression levels of cccDNA and pgRNA in HCC patients with occult HBV infection were similar or slightly lower in noncancerous tissues than in cancerous tissues, although more studies are needed to confirm these findings. However, the present results suggest that HBV persisted and was continuously replicated in cancerous and noncancerous cells.

Mutations in the S gene may alter HBsAg antigenicity and anti-HBs production. It was reported that a single amino acid mutation in the “a” determinant region (amino acids 124–147) of HBsAg could lead to a change in the immunologic epitope [28], and mutations or deletion in the S gene disrupted HBsAg production [29]. In the present study, two specific substitutions (I126T and I126V) were detected in occult HBV-infected patients. However, the HBV-DNA titre was <4 log copies/mL in all of the occult HBV-infected patients. Therefore, it is still unclear whether these substitutions are related to viral replication.

In conclusion, the present study confirmed that HBV persisted and was continuously replicated in cancerous and noncancerous tissues. In addition, cccDNA and pgRNA levels were thought to represent HBV replication in the liver and might contribute to the development of HCC in HBsAg carriers and occult HBV-infected patients.

## Figures and Tables

**Table 1:** Sequences and positions of primers and probes used in this study.

Primer and probe	Region	Position	Polarity	Sequence (5′ to 3′)	Reference
Occult case determination					
HBV 1	PreS-S	2815–2834	Sense	GGTCACCATATTCTTGGGAA	[6]
HBV 2	PreS-S	690–671	Antisense	AATGGCACTAGTAAACTGAG	[6]
HBV 17	PreS-S	3037–3057	Sense	AATCCAGATTGGGACTTCAA	[6]
HBV 4	PreS-S	459–440	Antisense	CCTTGATAGTCCAGAAGAAC	[6]
HBV 5	Precore-core	2021–2040	Sense	GCCTTAGAGTCTCCTGAGCA	[6]
HBV 6	Precore-core	2464–2448	Antisense	GTCCAAGGAATACTAAC	[6]
HBV 7	Precore-core	2048–2066	Sense	CCTCACCATACTGCACTCA	[6]
HBV 8	Precore-core	2385–2366	Antisense	GAGGGAGTTCTTCTTCTAGG	[6]
Pol 1 S	Polymerase	2412–2430	Sense	CGCGTCGCAGAAGATCTCA	[5]
Pol 1 AS	Polymerase	256–237	Antisense	CGAGTCTAGACTCTGTGGTA	[5]
Pol 2 S	Polymerase	2452–2476	Sense	GTATYCCTTGGACTCATAAGGTGGG	[5]
Pol 2 AS	Polymerase	2838–2814	Antisense	CTTGTTCCCAAGAATATGGTGACCC	[5]
X 1 S	X	1100–1121	Sense	CGCCAACTTACAAGGCCTTTCT	[5]
HBV 19	X	1550–1529	Antisense	CGTTCACGGTGGTCTCCAT	[6]
HBV 15	X	1380–1400	Sense	GCTAGGCTGTGCTGCCAACTG	[6]
HBV 21	X	1518–1497	Antisense	GGTCGGTCGGAACGGCAGACGG	[6]
Genotyping and amplification of “a” determinant region					
HB2F	S	414–433	Sense	TGCTGCTATGCCTCATCTTC	[7]
HB2R	S	989–970	Antisense	CATACTTTCCAATCAATAGG	[7]
Amplification of core promoter and precore regions					
HB7F	C	1611–1630	Sense	GAGACCACCGTGAACGCCCA	[7]
HB7R	C	2072–2048	Antisense	CCTGAGTGCTGTATGGTGAGG	[7]
cccDNA and pgRNA quantification					
CCC	X/C	1555–1573	Sense	GTGCCTTCTCATCTGCCGG	[8]
PGP	X/C	1826–1843	Sense	CACCTCTGCCTAATCATC	[8]
BC1	X/C	1974–1955	Antisense	GGAAAGAAGTCAGAAGGCAA	[8]
hbvLC	C	1874–1848	Antisense	GGAGGCTTGAACAGTAGGACATGAAC	[8]
hbvFL	C	1897–1876	Antisense	CYAAAGCCACCCAAGGCACAGC	[8]

**Table 2:** Clinical characteristics of patients.

	HBsAg-positive carriers (*n* = 14)	HBsAg−/HBcAb+ patients (*n* = 24)	*P* value
Male/female	13/1	21/3	NS
Age (years)	55.7 ± 12.0	68.7 ± 9.3	0.002
BMI	20.5 ± 3.7	23.1 ± 3.4	NS
Prevalence of BMI >22	3/14 (21%)	14/24 (58%)	0.03*
Platelet count (/mm^3^)	18.1 ± 4.0	19.3 ± 8.4	NS
Albumin (g/dL)	4.2 ± 0.6	3.9 ± 0.6	NS
AST (IU/L)	30.3 ± 5.9	74.0 ± 59.6	NS
HbA1c	5.04 ± 0.33	5.96 ± 0.87	<0.001
ICG-R 15 (%)	9.54 ± 2.76	11.15 ± 5.22	NS
Child-Pugh (A/B/C)	13/1/0	18/3/0	NS
HBV-DNA > 4 log copies/mL	6 (42.9)	0 (0.0)	0.012
Genotype B/C	1/13	0/17	NS
AFP > 200 (ng/mL)	7 (50%)	8 (33.3%)	NS
PIVKA-II > 400 (mAU/mL)	9 (64.3%)	14 (58.3%)	NS
Stage I/II/III/IV	1/4/2/3	1/6/2/9	NS

AST: aspartate aminotransferase; AFP: *α*-fetoprotein; PIVKA-II: protein induced by vitamin K absence or antagonist II; NS: not significant.

*Mann-Whitney's *U* test.

**Table 3:** Detection of HBV-DNA in 24 HCC patients with HBsAg-negative and HBcAb-positive patients.

	Cancerous tissue	Noncancerous tissue	*P* value
S region	9 (38%)	17 (71%)	0.020
Precore-core region	4 (17%)	4 (17%)	NS
Polymerase region	14 (58%)	10 (42%)	NS
X region	12 (50%)	21 (88%)	0.005
Positive in ≥2 regions	10 (42%)	20 (83%)	0.003

NS: not significant.

**Table 4:** The alignment of “a” determinant amino acid sequences.

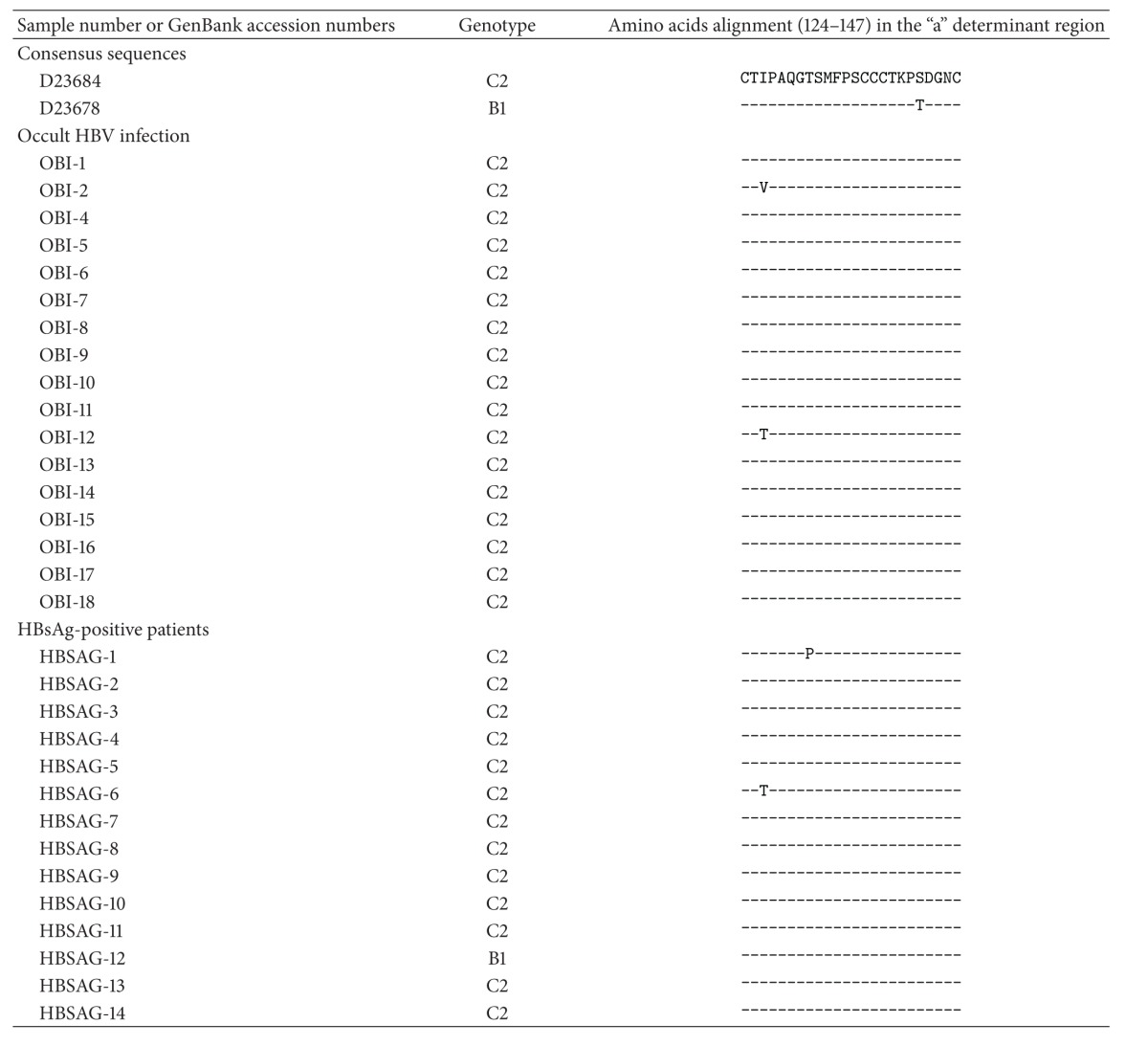

**Table 5:** Mutations in the core promoter and precore regions in patients divided by serological status.

	HBsAg-positive carriers (*n* = 14)	Occult HBV-infected patients (*n* = 9)	*P* value
T1753C	4 (29%)	0 (0%)	0.033
A1762T/G1764A	11 (79%)	5 (56%)	NS
T1846A	3 (21%)	3 (33%)	NS
G1896A	8 (57%)	4 (44%)	NS

NS: not significant.
